# Angiotensin (1-7) ameliorates the structural and biochemical alterations of ovariectomy-induced osteoporosis in rats via activation of ACE-2/Mas receptor axis

**DOI:** 10.1038/s41598-017-02570-x

**Published:** 2017-05-23

**Authors:** Hatem M. Abuohashish, Mohammed M. Ahmed, Dina Sabry, Mahmoud M. Khattab, Salim S. Al-Rejaie

**Affiliations:** 1 Department of Pharmacology and Toxicology, College of Pharmacy, King Saud University, Riyadh, Saudi Arabia; 2 Department of Biomedical Dental Sciences, College of Dentistry, University of Dammam, Dammam, Saudi Arabia; 3 Department of Medical Biochemistry and Molecular Biology, Faculty of Medicine, Cairo University, Cairo, Egypt; 4 Department of Pharmacology and Toxicology, Faculty of Pharmacy, Cairo University, Cairo, Egypt

## Abstract

The local and systemic renin angiotensin system (RAS) influences the skeletal system micro-structure and metabolism. Studies suggested angiotensin 1-7 (Ang(1-7)) as the beneficial RAS molecule via Mas receptor activation. This study examines the function of Ang(1-7) in bone micro-architecture and metabolism in an ovariectomized (OVX) rodent model of osteoporosis. OVX rats showed structural and bone metabolic degeneration in parallel with suppressed expressions of the angiotensin converting enzyme-2 (ACE-2)/Ang(1-7)/Mas components. The infusion of Ang(1-7) markedly alleviated the altered bone metabolism and significantly enhanced both trabecular (metaphyseal) and cortical (metaphyseal-diaphyseal) morphometry. Urinary and bones minerals were also improved in OVX rats by Ang(1-7). The infusion of the heptapeptide enhanced ACE-2/Mas receptor expressions, while down-regulated AngII, ACE, and AngII type-1 receptor (AT1R) in OVX animals. Moreover, Ang(1-7) markedly improved osteoprotegerin (OPG) and lowered receptor activator NF-κB ligand (RANKL) expressions. The defensive properties of Ang(1-7) on bone metabolism, structure and minerals were considerably eradicated after blockage of Mas receptor with A-779. Ang(1-7)-induced up-regulated ACE-2/Ang(1-7)/Mas cascade and OPG expressions were abolished and the expressions of ACE/AngII/AT1R and RANKL were provoked by A-779. These findings shows for the first time the novel valuable therapeutic role of Ang(1-7) on bone health and metabolism through the ACE-2/Mas cascade.

## Introduction

Diverse physiological and pathological actions of the renin angiotensin system (RAS) are now believed to be mediated through two axes. The first is the classical angiotensin converting enzyme (ACE)/angiotensin II (AngII)/angiotensin type-1 receptor (AT1R) axis responsible for RAS vascular constriction, proliferative and pro-inflammatory properties and the second is ACE-2/Ang(1-7)/Mas cascade, which usually counteracts on former axis effects^1^. Most of the heptapeptide Ang(1-7) reported actions are mediated by binding to the unique G protein receptor called Mas receptor^1^.

The relationship between RAS and bone health, structure and metabolism has been established and gained more attention lately, with various studies examining the role of RAS various components on bone density and fractures risks. The RAS is functionally active in several tissues not only systemically but also locally. Therefore, RAS expressions in bone microenvironments have been explored. Osteoblasts and osteoclasts express AT1R in cell cultures, which indicate the presence of local RAS in bone, while blockage of AT2R enhances bone mass^2^. Hirumaet al suggested that the local expression of RAS in bone might regulate bone remodeling and metabolism^3^. Hatton *et al*.^4^ demonstrated that adding AngII to osteoblast and osteoclast co-cultures may encourage bone resorption. Similar effect was reported in the same study with AngI and was attenuated moexiprilat (an ACE inhibitor), suggesting the production of AngII by ACE in bone cells from AngI^4^. Similarly, osteoblastic cell differentiation and bone formation were considerably suppressed by AngII in Schurman *et al*. *in vitro* study^5^. Binding of AngII to AT1R explained these effects^6^. These findings indicate that RAS components are present in adult bone.

AngII was found to accelerate osteoporosis via activation of osteoclastogenesis promoting factor, receptor activator NF-κB ligand (RANKL)^7^. Thus, counterbalancing AngII effects on bones might have a novel therapeutic value. Krishnan *et al*.^8^ study reported that Ang(1-7) reduces osteoclastogenesis process in a cell culture of bone marrow cells isolated from B6 mice tibias. In this study, addition of Ang(1-7) to the culture significantly reduced (by >50%) the number of multinuclear cells bearing tartrate-resistant acid phosphatase (TRAP^+^). Mas receptor was also found expressed in bone marrow-derived cells^1^. In addition, several medications known to be mediated their actions via stimulating ACE-2/Ang(1-7)/Mas receptor axis including ACEIs and AT1R blockers were reported to improve bone density and microstructure in several clinical and experimental studies^9–13^. Taken together, the current study examined the role of the heptapeptide (Ang(1-7)) in osteoporotic bone using the ovariectomized (OVX) Wistar rats as experimental model of osteoporosis.

## Results

### Effects on body and uterus weights

**Figure 1:**
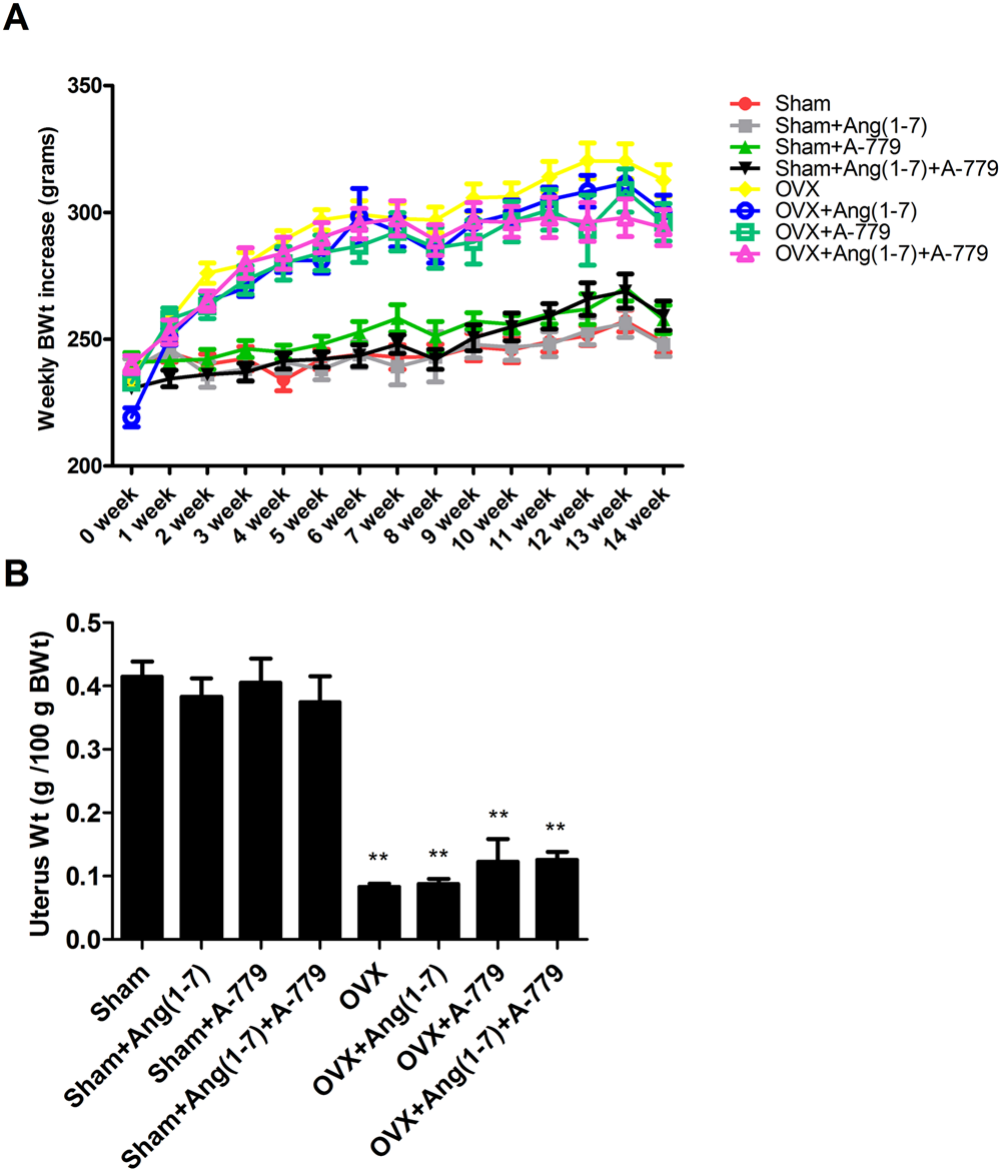
Effects of Ang(1-7) and/or A-779 treatments to sham and OVX animals for 6 weeks on (**A**) weekly body weights (BWt) increase and (**B**) uterus weights of Wistar albino rats. The mean weights of all OVX groups were compared to their respective sham groups in each week. The mean uterus dry weights per 100 g of final BWt of all OVX groups were compared to their respective sham groups. One-way ANOVA test followed by post hoc Student-Newman-Keuls multiple comparisons test were used for the statistical analysis. Columns and bars represent the mean ± SEM of each group (n = 8/group). Statistical significance were considered at *P < 0.05 and **P < 0.01.

The experiments were started using animals with a similar mean body weight (220–250 g). Starting from the third weeks of the experiment, all OVX animals exhibited a significant increase in their body weights compared to respective sham groups (Fig. 1). Neither Ang(1-7) and/or A-779 infusions for 6 weeks inhibited the body weight gain (Fig. 1). Uterus weights of all OVX animals were significantly decreased following 14 weeks of the OVX operation compared to respective sham groups (Fig. 1). Infusion of Ang(1-7) and A-779 alone or combined for 6 weeks did not prevent uterus atrophy (Fig. 1).

### Effects on bone metabolic turnover biomarkers

**Figure 2:**
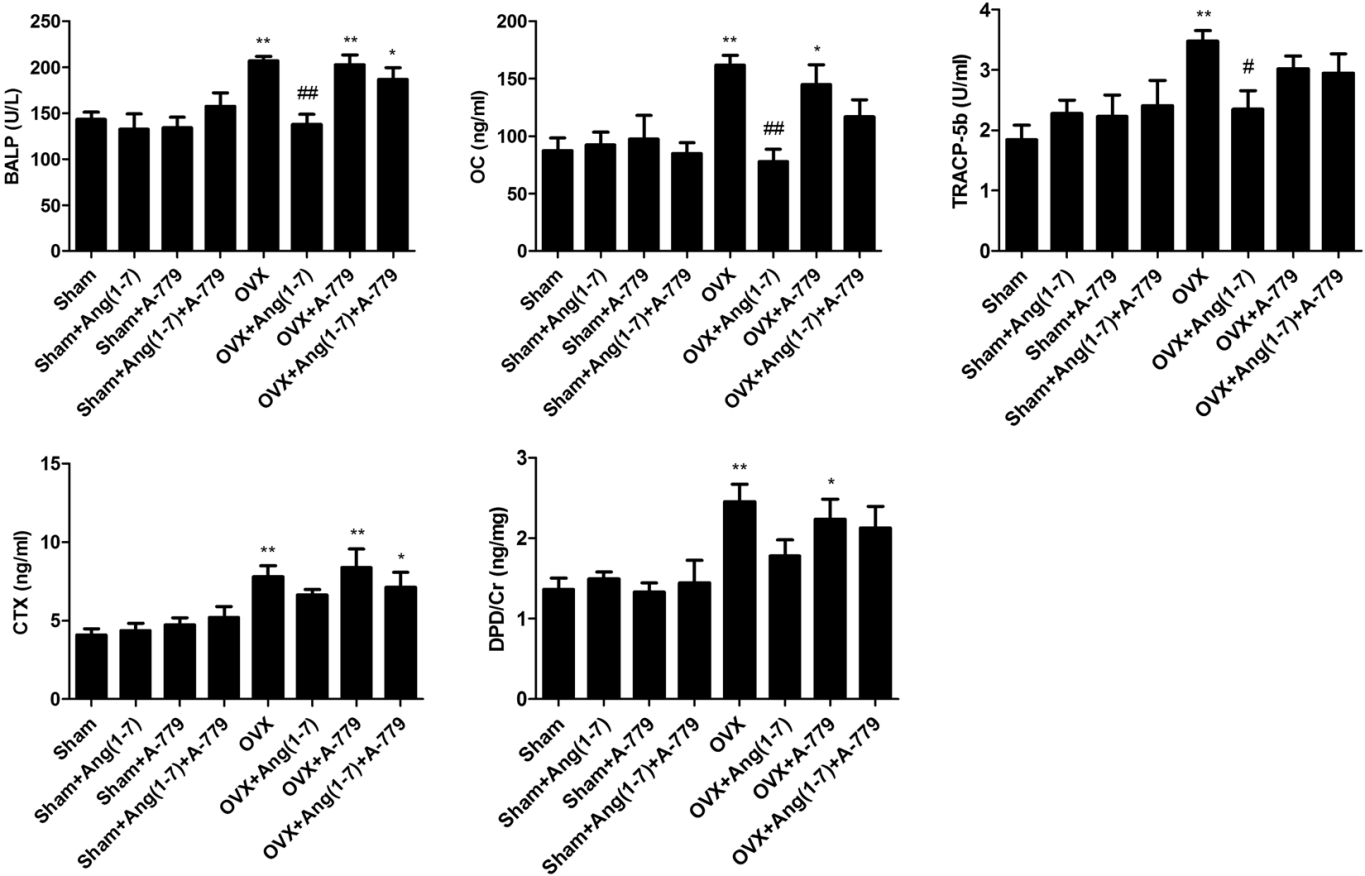
Effects of Ang(1-7) and/or A-779 treatments to sham and OVX animals for 6 weeks on bone turn over biomarkers in Wistar albino rats. Levels of BALP, OC, TRACP-5b and CTX were quantified in serum, while the urinary levels of DPD was estimated using sandwich ELISA technique. One-way ANOVA test followed by post hoc Student-Newman-Keuls multiple comparisons test were used for the statistical analysis. Columns and bars represent the mean ± SEM of each group (n = 8/group). Statistical significance was considered when *P < 0.05 and **P < 0.01 as compared to Sham group and ^#^P < 0.05 and ^##^P < 0.01 as compared to OVX group.

OVX group showed a considerable (P < 0.01) elevation in bone turnover biomarkers including the serum expressions of bone specific alkaline phosphatase (BALP), telopeptides of collagen type I (CTX), tartarate resistant acid phosphatase (TRAcP 5b), osteocalcin (OC) and urinary deoxypyridinoline (DPD) cross links compared to sham operated animals (Fig. 2). Ang(1-7) infusion to the OVX animals for 6 weeks significantly repaired BALP (P < 0.01), OC (P < 0.01) and TRACP-5b (P < 0.05) levels compared to OVX group (Fig. 2). However, infusion of A-779 markedly elevated serum BALP (P < 0.01), OC (P < 0.05), CTX (P < 0.01) and urinary DPD (P < 0.05) values (Fig. 2). Combining A-779 and Ang(1-7) infusions markedly eliminated the restorative effects of Ang(1-7), thus levels of all bone turnover biomarkers were not considerably different from the OVX groups. In addition, BALP and CTX levels in OVX + Ang(1-7)+A-779 group were significantly (P < 0.05) higher than sham group (Fig. 2).

### Effects on micro-CT structural integrity

**Figure 3:**
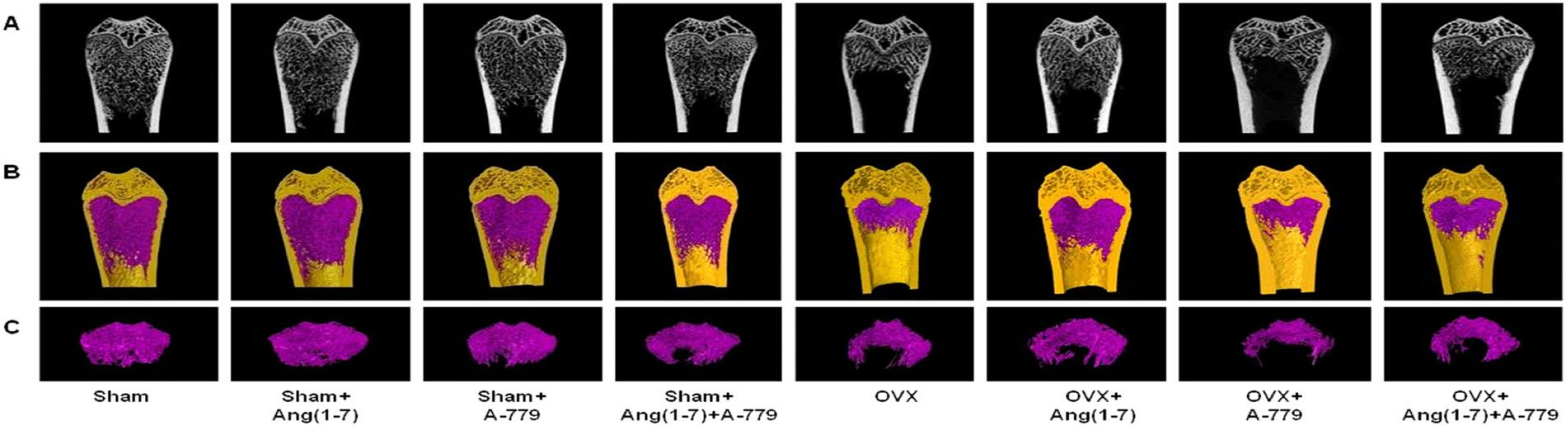
Representative (**A**) 2D and (**B**,**C**) 3D images of the femoral trabecular bone micro-architecture developed by micro CT. To analyze the trabecular bone micro-architecture, a volume of interest (VOI) with 1.6 mm height was selected starting 0.4 mm from the lowest end of the of the growth plate to the proximal end of the femur. (**A**) Sham; (**B**) Sham + Ang(1-7); (**C**) Sham + A-779; (**D**) Sham + Ang(1-7)+A-779; (**E**) OVX; (**F**) OVX + Ang(1-7); (**G**) OVX + A-779; (**H**) OVX + Ang(1-7)+A-779. Femur of the OVX group exhibited marked osteoporotic alterations in trabecular bone. Infusion of Ang(1-7) markedly attenuated these alterations, while A-779 abolished Ang(1-7) protective effects.

**Figure 4:**
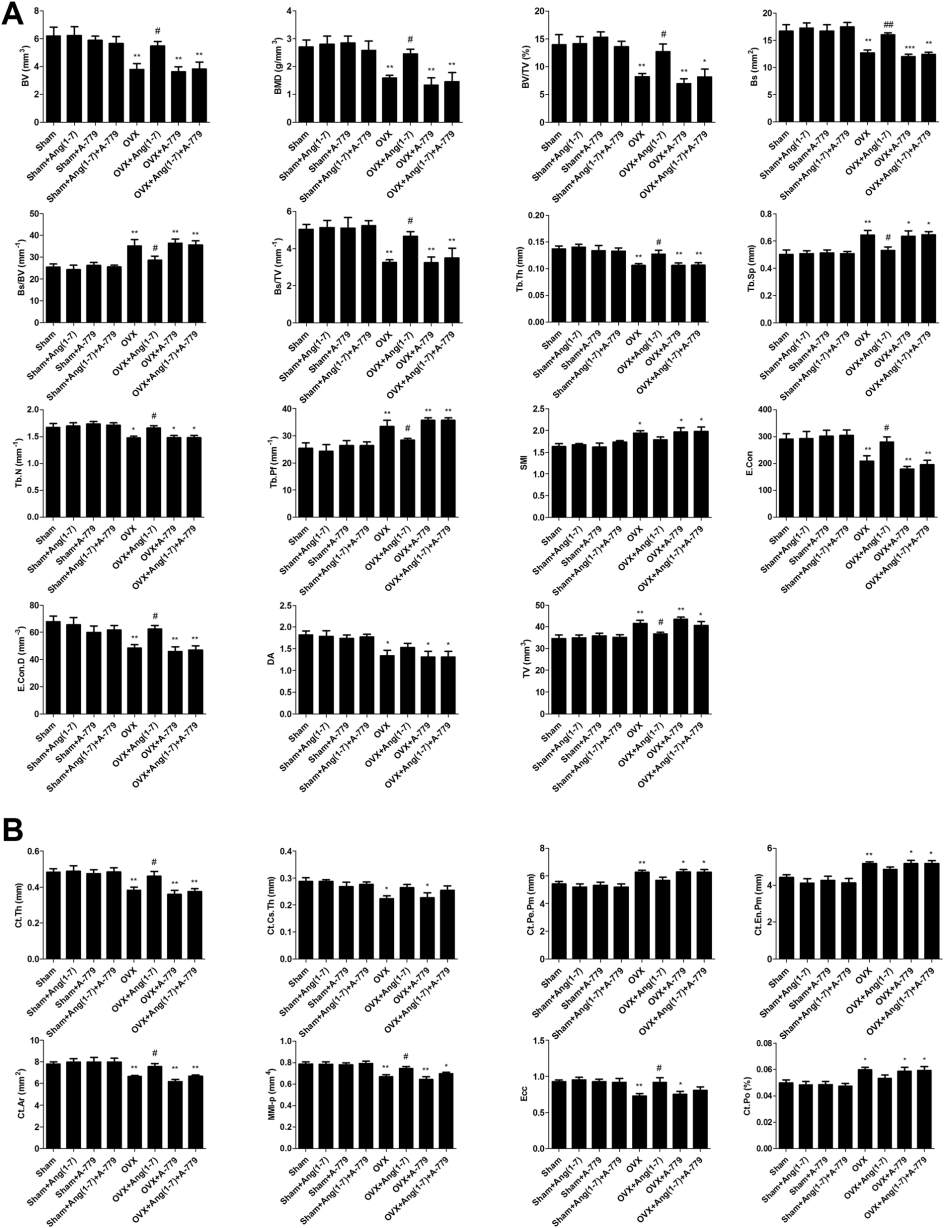
Effects of Ang(1-7) and/or A-779 treatments to sham and OVX animals for 6 weeks on distal femoral micro-architecture measured by micro-CT in Wistar albino rats. (**A**) Trabecular morphometric parameters in the compartment of the left distal femoral metaphyseal. (**B**) Cortical morphometric parameters in the metaphyseal-diaphyseal of left distal femoral bones. One-way ANOVA test followed by post hoc Student-Newman-Keuls multiple comparisons test were used for the statistical analysis. Columns and bars represent the mean ± SEM of each group (n = 8/group). Statistical significance was considered when *P < 0.05 and **P < 0.01 as compared to Sham group and ^#^P < 0.05 and ^##^P < 0.01 as compared to OVX group.

3D scanning and analysis of the Micro-CT developed images revealed significant trabecular and cortical loss in the distal femur following 14 weeks of OVX. Figure 3 shows generalized bone mass decrease in OVX group compared to sham group. The distal femur bone micro-architecture of the sham rats was not affected by Ang(1-7) and/or A-779 infusion (Fig. 3). Ang(1-7) infusion to OVX rats induced remission of the bone loss after 6 weeks (Fig. 3). The specific Mas receptor blocker (A-779) worsened OVX-induced bone micro-structure and removed the protective effects of Ang(1-7) (Fig. 3). The trabecular and cortical compartments of distal femoral metaphyseal and metaphyseal-diaphyseal were harmfully altered in OVX rats compared to sham group. There was a significant (P < 0.01) decrease in bone volume (BV), bone mineral density (BMD), bone volume/total volume ratio (BV/TV), bone surface (BS), bone surface/volume ratio (BS/TV), trabecular thickness (Tb.Th), trabecular number (Tb.N) (P < 0.05), euler connectivity (E.Con), euler connectivity density (E.Con.D) and degree of anisotropy (DA) (P < 0.05), while a significant (P < 0.01) increase in BS/BV, trabecular separation (Tb.Sp), trabecular pattern factor (Tb.Pf), structure model index (SMI) (P < 0.05) and TV in OVX animals (Fig. 4). Infusion of Ang(1-7) markedly (P < 0.05) corrected BV, BMD, BV/TV, BS (P < 0.01), BS/BV, BS/TV, Tb.Th, Tb.Sp, Tb.N, Tb.Pf, E.Con, E.Con.D and TV values as compared to OVX group (Fig. 4). Blocking of Mas receptor showed similar values of bone trabecular metaphyseal morphometric parameters compared to OVX group. Values of BV, BMD, BV/TV, BS, BS/TV, Tb.Th, Tb.N (P < 0.05), E.Con, E.Con.D and DA (P < 0.05) were significantly (P < 0.01) low, while BS/BV, Tb.Sp (P < 0.05), Tb.Pf, SMI (P < 0.05) and TV were significantly (P < 0.01) high in OVX + A-779 group as compared to sham control group (Fig. 4). Infusion A-779 with Ang(1-7) markedly abolished the protective effects of the peptide and showed no different trabecular compartment than OVX group (Fig. 4). On the other hand, cortical thickness (Ct.Th), cortical cross sectional thickness (Ct.Cs.Th) (P < 0.05), cortical cross sectional area (Ct.Ar), polar moment of inertia (MMI(p)) and eccentricity and cortical porosity (Ecc) were significantly (P < 0.01) reduced, while cortical periosteal perimeter (Ct.Pe.Pm), cortical endosteal perimeter (Ct.En.Pm) and cortical porosity (Ct.Po) (P < 0.01) were augmented in OVX group compared to sham (Fig. 4). Other sham operated animals infused with Ang(1-7) and A-779 alone or in combination did not show difference from sham control group in the cortical morphometric parameters (Fig. 4). Ang(1-7) significantly (P < 0.05) ameliorated OVX induced decrease in Ct.Th Ct.Ar MMI(p) Ecc (Fig. 4). For all cortical morphometric parameters, no significant difference was found between OVX animals treated with A-779 alone or in combination with Ang(1-7) and OVX untreated animals (Fig. 4). Adding A-779 to Ang(1-7) markedly eliminated its ameliorative effects on the lowered Ct.Th (P < 0.01), Ct.Ar (P < 0.01), MMI(p) (P < 0.05) and Ecc (P < 0.05) values (Fig. 4).

### Effects on bone weights and minerals levels

There was a significant (P < 0.05) decrease in left femoral dried bone weight in OVX animals compared to sham group (Table 1). Sham and OVX operated animals treated with Ang(1-7) and/or A-779 did not show significantly altered femoral bone weights as compared to sham and OVX control groups (Table 1). The increased femoral bone weights following Ang(1-7) infusion in OVX animals were not significant compared to OVX group (Table 1). Furthermore, ash weight of the untreated OVX animals and A-779 infused OVX animals were significantly (P < 0.05) lower than sham control group (Table 1). Other sham and OVX groups treated with Ang(1-7) and/or A-779 had no significant changed ash weights as compared to sham and OVX control groups (Table 1). Minerals concentrations in bones were markedly (P < 0.05) decreased in OVX animals including Ca^2+^ and P, while decrease in Mg^2+^ was not significant compared to sham group (Table 1). Infusion of the OVX animals with Ang(1-7) markedly (P < 0.05) corrected the decreased values of bone Ca^2+^ and P (Table 1). However, A-779 induced significant (P < 0.05) lower values of bone Ca^2+^. Bone Mg^2+^ levels were not markedly changed in sham or OVX animals with or without Ang(1-7) and/or A-779 infusions (Table 1). Serum levels of minerals were not markedly changed in OVX animals compared to sham group (Table 1). Infusion of Ang(1-7) and/or A-779 did not significantly change serum minerals concentrations in sham or OVX groups (Table 1). Urinary Ca^2+^, P and Mg^2+^ levels were markedly (P < 0.01, P < 0.05 and P < 0.01; respectively) increased in OVX untreated animals, while Ca^2+^ and P levels were significantly (P < 0.01 and P < 0.05 and respectively) increased in OVX + A-779 group compared to sham group (Table 1). Ang(1-7) infusion significantly (P < 0.05) attenuated OVX-induced increase in urinary minerals concentrations (P < 0.05, P < 0.01 and P < 0.01; respectively) (Table 1). Combining the Mas receptor blocker to Ang(1-7) markedly eliminated its minerals preservative effects against OVX-induced elevation (Table 1).

**Table 1:** Effects of Ang(1-7) and/or A-779 treatments to the sham and OVX animals for 6 weeks on the left femoral net bone and ash weights as well as minerals concentrations in femoral bones, serum and urine in Wistar albino rats.

	Sham	Sham + Ang(1-7)	Sham + A-779	Sham + Ang(1-7)+A-779	OVX	OVX + Ang(1-7)	OVX + A-779	OVX + Ang(1-7)+A-779
**Bone**								
Net Wt	277.7 ± 10.1	266.7 ± 11.7	265.2 ± 11.1	250.7 ± 10	224.1 ± 7.9*	248.8 ± 13.5	240.4 ± 6.4	234.4 ± 8.9
Ash Wt	54.7 ± 2.1	52.8 ± 1.3	51 ± 1.2	52.4 ± 1.7	46.4 ± 1.7*	50.2 ± 1.9	47 ± 1.6*	49.1 ± 1.2
Ca^2+^	32.4 ± 1.3	32.9 ± 1.1	32.8 ± 1.1	33.3 ± 1.3	27.8 ± 1*	32.1 ± 1.2^#^	28.2 ± 0.2*	30.3 ± 0.7
P	9.8 ± 0.2	9.6 ± 0.3	9.5 ± 0.5	9.5 ± 0.2	7.9 ± 0.4*	9.6 ± 0.4^#^	8.4 ± 0.5	8.4 ± 0.2
Mg^2+^	0.36 ± 0.01	0.36 ± 0.02	0.37 ± 0.01	0.37 ± 0.01	0.34 ± 0.0	0.36 ± 0.01	0.34 ± 0.02	0.35 ± 0.0
**Serum**								
Ca^2+^	1.2 ± 0.09	1.17 ± 0.09	1.18 ± 0.07	1.17 ± 0.06	1.14 ± 0.05	1.15 ± 0.08	1.08 ± 0.11	1.16 ± 0.08
P	0.78 ± 0.05	0.81 ± 0.04	0.80 ± 0.03	0.81 ± 0.02	0.76 ± 0.03	0.78 ± 0.02	0.75 ± 0.03	0.73 ± 0.03
Mg^2+^	0.06 ± 0.003	0.05 ± 0.005	0.06 ± 0.005	0.06 ± 0.004	0.05 ± 0.006	0.06 ± 0.003	0.05 ± 0.002	0.05 ± 0.004
**Urine**								
Ca^2+^	1.18 ± 0.06	1.36 ± 0.19	1.50 ± 0.14	1.36 ± 0.17	2.14 ± 0.22**	1.27 ± 0.20^#^	2.12 ± 0.22**	1.58 ± 0.14
P	1.51 ± 0.13	1.54 ± 0.20	1.55 ± 0.10	1.65 ± 0.21	2.76 ± 0.33*	1.05 ± 0.35^##^	2.50 ± 0.29*	1.86 ± 0.37
Mg^2+^	0.92 ± 0.22	1.66 ± 0.41	1.85 ± 0.36	1.72 ± 0.16	2.67 ± 0.38**	0.68 ± 0.07^##^	1.92 ± 0.22	1.46 ± 0.28

The net bone weights were expressed as mg/100 g body weight, while ash weights were expressed as mg/100 mg net bone weight. The concentrations of minerals including Ca^2+^, P and Mg^2+^ were measured by ICP-MS and expressed as mg/100 mg Ash in bone, mmol/L in serum, and mmol/mmol creatinine in urine. One-way ANOVA test followed by post hoc Student-Newman-Keuls multiple comparisons test were used for the statistical analysis. Data are expressed as mean ± SEM of each group (n = 8/group). Statistical significance was considered when *P < 0.05 and **P < 0.01 as compared to Sham group and ^#^P < 0.05 and ^##^P < 0.01 as compared to OVX group.

### Effects on RAS proteins expression and osteoclastogenesis modulating factors (OPG/RANKL)

**Figure 5:**
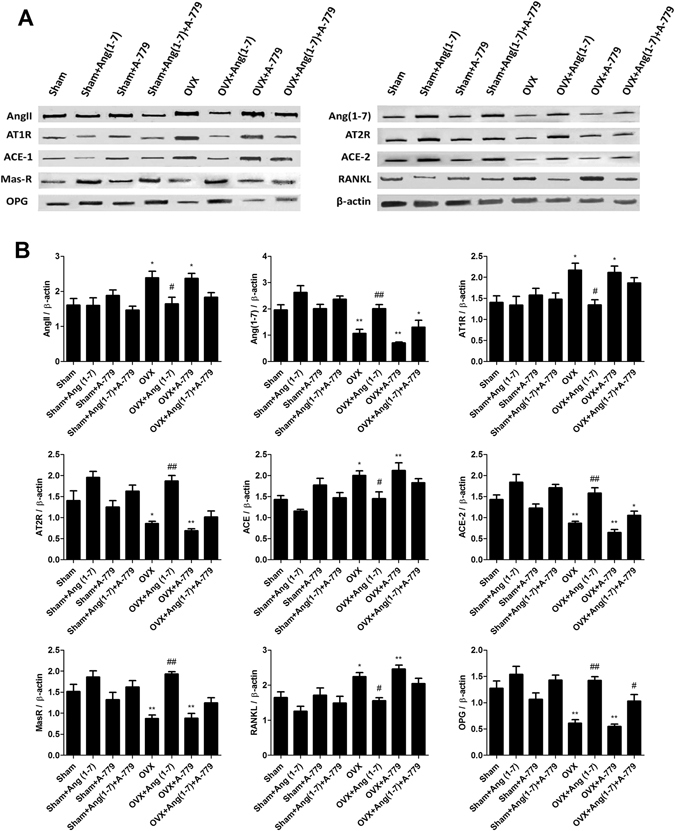
Effects of Ang(1-7) and/or A-779 treatments to the sham and OVX animals for 6 weeks on the expressions of RAS different proteins and osteoclastogenesis modulating factors in the femoral bone heads. (**A**) Western blot analysis bands showing the expressions of AngII, Ang(1-7), AT1R, AT2R, ACE, ACE-2, MasR, RANKL and OPG. (**B**) Quantification of the scanning densitometry of the western blot bands (n = 8/group) expressed as arbitrary units. One-way ANOVA test followed by post hoc Student-Newman-Keuls multiple comparisons test were used for the statistical analysis. Columns and bars represent the mean ± SEM of each group. Statistical significance was considered when *P < 0.05 and **P < 0.01 as compared to Sham group and ^#^P < 0.05 and ^##^P < 0.01 as compared to OVX group.

Protein analysis of the animals femoral heads revealed that OVX animals had a significant increase in AngII (P < 0.05), AT1R (P < 0.05), ACE (P < 0.05) and RANKL (P < 0.05) expressions, while the expressions of Ang(1-7) (P < 0.01), AT2R (P < 0.05), ACE-2 (P < 0.01), Mas receptor (P < 0.01) and OPG (P < 0.01) were significantly decreased compared to sham group (Fig. 5). Sham operated groups infused with Ang(1-7) and/or A-779 did not show significantly different values of AngII, Ang(1-7), AT1R, AT2R, ACE, ACE-2, Mas receptor, RANKL and OPG expressions as compared to sham control group, with exception of AT2R and ACE-2 expressions in Sham + Ang(1-7) group (P < 0.05) (Fig. 5). Conversely, OVX animals with Ang(1-7) infusion had considerable lower (P < 0.05) expressions of AngII, AT1R, ACE and RANKL and higher (P < 0.01) expressions of Ang(1-7), AT2R, ACE-2, Mas receptor and OPG compared to OVX group (Fig. 5). Experimental blocking of the Mas receptor by A-779 in the OVX animals did not change AngII, Ang(1-7), AT1R, AT2R, ACE, ACE-2, Mas receptor, RANKL and OPG proteins expressions in relation to OVX group, while AngII (P < 0.05), AT1R (P < 0.05), ACE (P < 0.01) and RANKL (P < 0.01) expressions were significantly higher and Ang(1-7), AT2R, ACE-2, MasR and OPG were significantly (P < 0.01) lower than sham group (Fig. 5). Combining A-779 infusion with Ang(1-7) markedly abolished the effects of the peptide, where OVX + Ang(1-7)+A-779 group showed no significant different expressions of AngII, Ang(1-7), AT1R, AT2R, ACE, ACE-2, Mas receptor and RANKL compared to OVX group (Fig. 5).

## Discussion

ACE-2/Ang(1-7)/Mas receptor cascade is suggested to be the beneficial arm in the biological effects of systemic and local RAS^14^. In the existing study, Ang(1-7) improved the disturbed bone metabolism and micro-architecture. Furthermore, Ang(1-7) markedly enhanced the mineralization process and attenuated osteoclastogenesis. Blocking of the G-protein coupled receptor (Mas) by A-779 markedly abolished Ang(1-7) favorable effects on bone health suggesting the vital role of Mas receptor in mediating Ang(1-7) osteo-protective effects.

OVX rat model is a well-known and FDA recommended model for osteoporosis^15^. The increase in body weights and uterine atrophy of OVX animals verified the success of the model in the current study. The significant increase in body weights in OVX groups is in agreement with several experimental studies using the same model^16–19^. It is reported that estrogen deficiency results in body weight gain and fats redistribution via altering leptin functions or by increasing food desire^20^. One study reported that estrogen administration after menopause may reduce food intake, which may be regulated by estrogen receptor-α (ER-α)^21^. We did not find a significant decrease in body weight in OVX animals after Ang(1-7) administration in the current study. In contrast with our study, ACE-2 activation reduced fat deposition and lipogenesis^22^, and Ang(1-7) treatment decreased body weights and fat mass of rodents fed on fat rich diets^23, 24^. Different experimental models of body weight gain may explain such contradictions. Therefore, further studies are recommended to relate Ang(1-7) effects on fat distribution and adipogenesis during menopause. On the other hand, estrogen deficiency markedly induced uterine atrophy, which was not prevented by Ang(1-7). Although, Ang(1-7) and Mas receptor are expressed in uterus tissues, they did not regulate uterus weight in lack of estrogen^25^.

Products of bone metabolism are considered a valuable tool to monitor the prognosis of osteoporosis and the therapeutic effectiveness of medications. In the present study, the levels of bone metabolic biomarker including BALP, OC, TRAcP, CTX and DPD were studied as an indication for bone metabolism. Although, BALP is produced in the osteoblasts, where it plays a essential role in bone matrix calcification^26^, its serum concentration usually is found elevated in case of excessive bone loss^27^. Serum OC levels also increase in osteoporosis as it has high affinity to minerals in bones^28^. Furthermore, TRAcP is bone resorbing enzyme found in ruffled border area of the osteoclasts^29, 30^. Bone resorption results in fragmentation of collagen to several products such as CTX telopeptides and DPD (a very specific cross link in bone collagen) both of which are released in to blood during bone metabolism^31, 32^. Taken together, the concentrations of these metabolic biomarkers were increased in our model of osteoporosis. However, infusion of Ang(1-7) corrected the elevated biomarkers levels suggesting alleviated bone metabolism and osteoclastic activates. This comes in agreement with Krishnan *et al*.^8^ findings, where Ang(1-7) showed suppressive effects on the osteoclastogenesis process. Ang(1-7) effects on bone metabolism in current study were regulated by Mas receptor, since blocking of the receptor by A-779 in OVX animals elevated the metabolic markers again.

Femoral bone micro-environment was considerably altered in OVX rats. Measurement of bone morphometry in metaphyseal region revealed a weakened trabecular thickness, number and connections along with decreased density. Cortical bone in the metaphyseal-diaphyseal region was also altered with decreased connections and increased porosity. It is well-established that estrogen controls the bone resorping osteoclasts numbers and activities via the ER-α, which induces their apoptosis and reduces their life span through regulation of the FasL gene^33^. This may explain the alterations in trabecular and cortical micro-environment noticed in OVX animals. Micro-CT analysis revealed that Ang(1-7) can improve trabecular and cortical bone weakness and thinning and increase bone density. In consistent with Nie *et al*. study that reported Mas receptor expression in the osteoclasts precursors^1^, we reported that Ang(1-7) effects on trabecular and cortical metaphyseal and diaphyseal are abolished by Mas receptor occlusion suggesting a local physiological role of Ang(1-7) via Mas receptor dependant pathway

50–70% of normal bone are minerals (mainly Ca^2+^ and P) in nature and the rest are organic materials. Bone mineralization process is a complex biological process at which minerals are deposited down on bone matrix. It requires a crosstalk between the skeletal tissue and circulation as well as kidneys with several promoting or inhibiting factors^34^. Mineral concentrations were also markedly affected in OVX animals. Ca^2+^ and P excretions were increased in OVX animals, while their bone concentrations were reduced, such effect which confirms the provoked osteoclasts functions in the estrogen deficient rats. Ang(1-7) markedly attenuated minerals renal loss and enhanced their deposition in bones, while A-779 eliminated Ang(1-7) effects. Ang(1-7) effects on bone mineralization process may be systemic or local. ACE-2/Ang(1-7)/Mas receptor cascade is recognized to improve endothelial functions in different cardiovascular disorders^35^. Osteoporosis was found to be associated with endothelial dysfunction^36^ and the vaso-preservative agents were found to enhance calcification and bone health^37, 38^. Therefore, it may be proposed that bone minerals resolved by Ang(1-7) were mediated by its vaso-protective properties. In addition, we advocate that the local Ang(1-7) might inhibit the osteoclastic demineralization via Mas receptor, thus promoting the local mineralization process.

Expressions of different RAS components were reported in the skeletal systems in the current investigation in accordance with other reports^2, 39, 40^. The effector member of RAS (AngII) was found highly expressed together with its forming enzyme, ACE, and receptor (AT1R) in femoral bones heads of OVX osteoporotic animals, which comes in harmony with several studies suggesting the induction of osteoporosis via activation of classical RAS pathway (ACE/AngII/AT1R). Ang II was found in one study to stimulate bone loss and osteoclasts activity via triggering RANKL^7^. Asaba *et al*. also demonstrated that AngII can exacerbate the osteoporotic conditions^39^. Inactivation of ACE was also found to improve bone mass in clinical and experimental studies^10, 39, 41, 42^. Moreover, blocking of AT1R showed therapeutic and protective values on the skeletal tissues^7, 11, 43, 44^. The conventional ACE/AngII/AT1R pathway was also reported to be involved in different experimental bone diseases including age-related osteoporosis in mice^45^, bone deteriorations following obstructive nephropathy^46^ or type 1 diabetes^47^, postmenopausal osteoporosis in OVX animals^7, 10^ and glucocorticoid-induced osteoporosis^48^. Therefore, it could be suggested that opposing action of conventional ACE/AngII/AT1R pathway may boost bone health and structure, which was reported in the present study as Ang(1-7) treatment markedly down-regulated ACE/AngII/AT1R expression and improved bone metabolism, structure and mineralization.

On the other hand, the expression of ACE-2/Ang(1-7)/Mas cascade was found down-regulated in estrogen deficient rats. Ang(1-7) administration to the OVX animals was associated with improved bone health and higher expressions of ACE-2/Ang(1-7)/Mas receptor cascade, while blocking of the beneficial cascade by A-779 altered their expressions along with bone structure and metabolism. Bone resorption is highly induced by the membrane-bound secreted protein (RANKL) and OPG is known to oppose such effect^49^. Interestingly, the expression of RANKL was augmented, while OPG expression was reduced in OVX animals indicating bone resorption and loss. However, the induction of ACE-2/Ang(1-7)/Mas cascade was markedly linked with attenuation of the osteoclastogenesis process indicated by down-regulating RANKL and up-regulating OPG expressions in bone tissue. These findings were similar to Krishnan *et al*. study at which Ang(1-7) inhibited osteoclastogenesis process in bone marrow cells isolated from B6 mice tibias and significantly decreased in the number of TRAP^+^ multinuclear cells (by >50%). Although AT2R expression was declined in OVX rats and augmented with Ang(1-7) treatment. Reports demonstrated that blockade of this receptor may increase bone mass^2^. Therefore, we suggest that osteo-preservative effects of heptapeptide (Ang(1-7)) were mediated via its Mas receptor especially that blockage of the receptor markedly increased RANKL expression and negatively reshaped bone structure and metabolism.

Finally, the present documented osteo-preservative effects of Ang(1-7) could suggest a new therapeutic approach of osteoporosis. The synthesis and identification of a non-peptide compound that mimics Ang(1-7) action might represent a new treatment for the metabolic disorder. AVE-0991 (AVE) (5-formyl-4-methoxy-2-phenyl-1-[[4-[2-ethyl-aminocarbonyl sulfonamido-5-isobutyl-3- thienyl]-phenyl]-methyl]-imidiazole) is a synthetic compound that acts as Mas receptor agonist and reproduced the anti-fibrotic, anti-proliferative and anti-inflammatory effects of Ang(1-7) in previous reports^50, 51^. Thus, whether the biological active new compound (AVE) would have an osteo-protective effect similar to the heptapeptide, Ang1-7, needs to be further investigated and elucidated on the skeletal system.

## Conclusion

It could be concluded that ACE-2/Ang(1-7)/Mas receptor cascade may act as the beneficial player in RAS to eliminated deleterious effects of the conventional ACE/AngII/AT1R pathway on the skeletal tissues. The identification of Ang(1-7) osteo-preserving properties may open the door for new therapeutic agents and further understanding of the bone metabolic disorders.

## Methods

### Chemicals

Angiotensin I/II (1-7) trifluoroacetate salt (Ang(1-7)) (Cat #H-1715) and the specific Mas receptor antagonist (D-Ala^7^)-Angiotensin I/II (1-7) trifluoroacetate salt (A-779) were purchased from Bachem AG (Hauptstrasse, Bubendorf, Switzerland). Alzet osmotic pumps (model 2006) were purchased from Durect Corporation (Minneapolis, USA). The diagnostic ELISA kits specific for rat BALP, CTX, TRAcP-5b, OC and DPD cross links were bought from Biotang Inc, (Waltham, Massachusetts, USA). The primary antibodies of AngII (Cat #sc-9040), Ang(1-7) (Cat #sc-319824), Mas-receptor (Cat #sc-54848), RANKL (Cat #sc-9073) and OPG (Cat #sc-8468) were supplied from Santa Cruz Biotechnology, Inc. (Dallas, Texas, USA), while the primary antibodies for AT1R (Cat #NBP1-77078), AT2R (Cat #NBP1-77368), ACE (Cat #NBP1-19760), ACE-2 (Cat #NBP1-76614PEP) and the secondary antibody (Goat antirabbit IgG- HRP-1mg Goat mab) were purchased from Novus Biologicals (Littleton, Colorado, USA).

### Animals usage and ethical approval

In the existing study, similar aged female Wistar rats weighting approximately 220–250 g were supplied from Experimental Animal Care Center at college of Pharmacy, King Saud University (KSU). Animals were subject to standard and controlled experimental conditions. This study was in accordance with the National Institute of Health Guidelines (NIH Publications No. 80-23; 1996). This study was ethically accepted by the Research Ethical Committee, College of Pharmacy, KSU as well as the ethical committee, Faculty of Pharmacy, Cairo University, Cairo, Egypt (ethical approval No. PT-208).

### The experimental model and study protocol

A bilateral OVX operation was use to induce postmenopausal osteoporosis in female Wistar albino rats. The animals were generally anesthetized using single IP injection of ketamine + xylazine combination (80 mg/kg and 5 mg/kg; respectively). After ligation, the ovaries were excised from a longitudinal incision made on the dorsolateral body region. Sham rats had the same procedure without the ligation and excision. The risk of postoperative infection was eliminated by applying fusidic acid topical antibiotic cream twice weekly for 4 weeks. Following the sham and OVX operations, animals were divided into eight groups (n = 8/group) including, Sham, Sham + Ang(1-7), Sham + A-779, Sham + Ang(1-7)+A-779, OVX, OVX + Ang(1-7), OVX + A-779 and OVX + Ang(1-7)+A-779. Eight weeks following sham and OVX operations, osmotic pumps (model 2006, Alzet, Durect Corporation, Minneapolis, USA) were implanted subcutaneously to deliver Ang(1-7) (200 ng/kg/min), A-779 (400 ng/kg/min) or a mixture of Ang(1-7) and A-779 (200 ng/kg/min and 400 ng/kg/min; respectively) for 6 consecutive weeks^52^. Body weights of all animals were monitored and recorded at the beginning and every week throughout the study. Animals’ general health was maintained during periods of treatments. At the end of treatments periods, animals were placed fasting in metabolic cages for 16 hr and urine samples were collected and frozen at −70 °C. Then, the blood samples were collected by cardiac puncture under ketamine + xylazine anesthesia and serum samples were obtain by centrifugation at 4000 RPM to. Animals were then sacrificed by decapitation and the uterine tissues and femoral bones were removed and cleaned. Uterine tissues were cleaned from fats and the wet weights were determined directly and expressed as g/100 g body weight. In each rat, both femurs were removed and cleaned from soft tissues. The left femoral bone samples were frozen at −70 °C until analyzed, while the right femoral bones were kept in 10% formalin for mico-CT and minerals analysis.

### Measurement of bone turnover biomarkers

The levels of bone metabolic biomarkers including BALP, OC, TRACP-5b and CTX were assayed in serum, while the urinary levels of DPD was also quantified using a rat solid phase-phase sandwich ELISA kit (BiotangInc, Waltham, Massachusetts, USA). Results of DPD levels in urine were normalized using creatinine concentration (mg/dl).

### Determination of femoral bone morphometry using micro-CT

Trabecular and cortical bone micro-architecture were determined by measuring the morphometric parameters in the distal right femoral bones of the animals. High-resolution *in*-*vivo* micro-CT scanner (Skyscan 1176 Bruker micro-CT, Kartuizersweg 3B, 2550 Kontich, Belgium) was used for samples scanning. Scanning conditions were as follow; X-ray voltage: of 70 kV, X-ray current: 143 μA, Filter: 1 mm aluminium, Image pixel size: 6–8 μm. Reconstruction was performed using the SkyscanNrecon software. The trabecular (metaphyseal) and cortical (metaphyseal-diaphyseal) bones were selected in reference to a previously identified growth plate. A cross-sectional slice was selected as a growth plate reference slice that can be identified in all micro-CT scans of the distal femur. The selection of the regions of interest (ROI) based on a reference growth plate. The cortical and trabecular regions identified as areas within the femur long axis relative to the reference growth plate. To analyze the trabecular bone micro-architecture, a 1.6 mm height volume of interest (VOI) was chosen starting at the lowest end of the of the growth plate (0.4 mm) in the direction of the proximal end of the femur. The cortical region commenced about 3.08 mm from the growth plate and extended for a further 0.77 mm towards the metaphysic direction. The 3D and 2D trabecular and cortical morphometric parameters were calculated for selected ROIs.

### Quantification of serum, urine and femoral bone minerals concentrations

The dried right rat femoral bones of both sham and OVX animals were weighted and the weight was expressed in grams. After weighing, the femoral bones were subjected to ashing in a muffle furnace (Lenton thermal designs, Parsons Lane, Hope, Hope Valley, S33 6RB, UK) for 16 hr at 700 °C. Next, the ashed bones were weighed and the weights were expressed as mg/100 mg net bone weight. Samples were then digested in 70% nitric acid (5 ml per each 100 mg ash) and the acidic mixtures were incubated shaking in a water bath (37 °C) for an overnight. Samples were then diluted with deionized distilled water (1:9 ratio)^16, 53^. On the other hand, 0.1 ml of serum and urine samples were directly digested with 0.9 ml 70% nitric acid, then, diluted with deionized distilled water (1:9 ratio). The diluted ash, serum and urine samples were used to quantify the concentrations of calcium, inorganic phosphorus and magnesium (Ca^2+^, P and Mg^2+^) by an inductive coupled plasma mass spectroscopy (ICP-MS) (PerkinElmer Life and Analytical Sciences 710 Bridgeport Avenue Shelton, CT, USA). Minerals concentrations in the femoral bones were expressed as mg mineral per 100 mg ash, while minerals concentrations in serum and urine were expressed as mmol/L. Urine results were normalized using creatinine concentration (mmol/L).

### Quantitative Western blot for bone proteins

The Ready Prep™ protein extraction kit (Cat #163-2086, Bio-Rad inc., CA, USA) was employed for protein extraction procedure from the epiphysis of the bone tissue (30 mg) using an ultrasonic probe. Bradford protein assay kit (Cat #SK3041, Bio basic inc., Ontario, Canada) was used for quantitative protein analysis. Samples proteins were then separated on a polyacrylamide gel according to their molecular weight in a standard migration running buffer. 20 µg of total protein was loaded per each mini-gel well. The separation was visualized using stain-free technology and ChemiDoc TM imager. The gel was then assembled in transfer sandwich with a PVDF membrane. The sandwich was placed in the transfer tank with 1x transfer buffer composed of 25 mMTris and 190 mM glycine and 20% methanol. Then, the blot was run for 7 min at 25 V to allow protein bands transfer from gel to membrane using BioRad Trans-Blot Turbo. The blot separation was visualized and imaged immediately using stain-free blot technology and ChemiDoc TM imager. The membrane was blocked in tris-buffered saline with Tween 20 (TBST) buffer and 3% bovine serum albumin (BSA) for 1 hr at room temperature. The primary antibodies of AngII (sc-9040), Ang(1-7) (sc-319824), ACE (NBP1-19760), ACE-2 (NBP1-76614PEP), AT1R (NBP1-77078), AT2R (NBP1-77368), Mas-receptor (sc-54848), RANKL (sc-9073) and OPG (sc-8468) were diluted in TBST according to manufactured instructions. Incubation was done overnight in each primary antibody solution, against the blotted target protein, at 4 °C in the HRP-conjugated secondary antibody (Goat anti-rabbit IgG- HRP-1mg Goat mab -Novus Biologicals) solution for 1 hr at room temperature. The chemiluminescent substrate (Clarity™ Western ECL substrate Bio-Rad cat #170-5060) was applied to the blot according to the manufacturer’s recommendation. Image analysis software was used to read the band intensity of the target proteins against control sample beta actin (housekeeping protein) by protein normalization on the ChemiDoc MP imager.

### Statistical Analysis

Results of the current study were presented as mean ± standard error (SEM). The statistical analysis was carried out by one-way analysis of variances (ANOVA) followed by multiple comparison post hoc test (Student-Newman-Keuls). Mean differences between groups were reflected as significant when P value is equal to or less than *0.05 and **0.01 Vs Sham group as well as ^#^0.05 and ^##^0.01 Vs OVX group. Graph pad prism (Graph-pad prism (version no. 5) Inc., La Jolla, CA, USA) was employed as statistical analyzing software.
